# Risk of Oral Human Papillomavirus Infection Among Sexually Active Female Adolescents Receiving the Quadrivalent Vaccine

**DOI:** 10.1001/jamanetworkopen.2019.14031

**Published:** 2019-10-25

**Authors:** Nicolas F. Schlecht, Martin Masika, Angela Diaz, Anne Nucci-Sack, Anthony Salandy, Sarah Pickering, Howard D. Strickler, Viswanathan Shankar, Robert D. Burk

**Affiliations:** 1 Department of Cancer Prevention and Control, Roswell Park Comprehensive Cancer Center, Buffalo, New York; 2 Department of Epidemiology & Population Health, Albert Einstein College of Medicine, Bronx, New York; 3 Mount Sinai Adolescent Health Center, Department of Pediatrics, Icahn School of Medicine at Mount Sinai, Manhattan, New York; 4 Department of Pediatrics, Division of Genetics, Albert Einstein College of Medicine, Bronx, New York; 5 Department of Microbiology & Immunology, Albert Einstein College of Medicine, Bronx, New York; 6 Department of Obstetrics & Gynecology and Women’s Health, Albert Einstein College of Medicine, Bronx, New York

## Abstract

**Importance:**

Human papillomavirus (HPV) is the most common sexually transmitted infection in the United States, and oral HPV infection is associated with increased risk of oropharyngeal cancer.

**Objective:**

To describe the risk factors for oral HPV in sexually active female adolescents receiving the quadrivalent vaccine.

**Design, Setting, and Participants:**

Longitudinal cohort study involving repeated collection of oral rinse specimens from sexually active female adolescents conducted between October 19, 2007, and March 9, 2017, at a large adolescent health center in New York, New York, that provides free health care, including HPV vaccination.

**Exposures:**

Human papillomavirus vaccination and self-reported history of sexual behavior.

**Main Outcomes and Measures:**

Prevalence of HPV in the oral cavity.

**Results:**

Among the 1259 participants who were included in this study, median age at entry into the study was 18 (range, 13-21) years; 638 (50.7%) were of African American descent, 569 (45.2%) were of Hispanic descent, 43 (3.4%) reported another race/ethnicity, and race/ethnicity was unspecified for 9 (0.7%). The median (mode) age at first sexual activity was 14.8 (14) years, and 1161 (92.2%) reported having had oral sex. Human papillomavirus DNA was detected in baseline oral rinse samples of 78 of the 1259 participants (6.2%; 95% CI, 4.9%-7.6%). There was a significant decrease in oral HPV detection with time (in years) since first engaging in sexual activities, independent of age and concurrent detection of cervical HPV; comparing 4 or more years with 1 year or less, the odds ratio was 0.45 (95% CI, 0.21-0.96). Detection of vaccine types (HPV-6, HPV-11, HPV-16, and HPV-18) was significantly lower among participants who had received at least 1 dose of the quadrivalent HPV vaccine at the time of enrollment compared with those who were unvaccinated (odds ratio, 0.20; 95% CI, 0.04-0.998).

**Conclusions and Relevance:**

This study’s findings suggest that detection of HPV in the oral cavity is not uncommon in sexually active female adolescents. In addition, HPV vaccination is associated with a significant decrease in detection of HPV vaccine types in the oral cavity.

## Key Points

**Question:**

What is the prevalence of oral human papillomavirus (HPV) in sexually active female adolescents receiving the quadrivalent vaccine?

**Findings:**

This longitudinal cohort study of female adolescents detected HPV DNA in 6.2% of oral rinse samples, with prevalence decreasing with time since initiation of sexual activity. Detection of HPV types targeted by the quadrivalent vaccine (HPV-6, HPV-11, HPV-16, and HPV-18) was also significantly lower among those who received at least 1 dose of vaccine than among unvaccinated participants.

**Meaning:**

This study’s findings suggest that detection of HPV in the oral cavity is not uncommon in sexually active female adolescents, but that HPV vaccination is associated with a significant decrease in detection of HPV types in the oral cavity.

## Teaser

This cohort study assesses associations between sexual activity, the oral presence of human papillomavirus (HPV), and HPV vaccination among sexually active female adolescents vaccinated against 4 HPV types.

## Introduction

The human papillomavirus (HPV) is the most common sexually transmitted infection in adolescent and young adult women and is responsible for the development of almost all cervical cancers and most anal and oropharyngeal cancers.^1,2^ Recent reports indicate that from 1988 to 2004, oropharyngeal cancers increased more than 200% in the United States.^3^

A recent study of the US National Health and Nutrition Examination Survey (NHANES) showed a bimodal distribution in the prevalence of oral HPV in women, with a first peak between ages 20 and 25 years at 4.8% for any mucosal HPV type.^4^ The prevalence of oncogenic HPV types, including HPV-16, which is found in the majority of HPV-positive oropharyngeal cancers, differed among women of different racial/ethnic backgrounds.^5^ Increasing rates of HPV-associated oropharyngeal cancers suggest that additional research is warranted.^3^

A previous study reported a decline in cervical and anal HPV infection after vaccination in sexually active female adolescents in New York, New York.^6^ Human papillomavirus vaccination has also been associated with decreased detection in the oral cavity of HPV types targeted by the vaccine.^7,8^ In the present study, we investigated the association of HPV vaccination with HPV prevalence in the oral cavity in a cohort of sexually active female adolescents.

## Methods

### Study Population

We tested for oral HPV DNA in oral rinse samples collected from a prospectively enrolled cohort of 1273 patients attending a large adolescent health clinic in New York City offering free health care and HPV vaccination between October 19, 2007, and March 9, 2017; follow-up continued to April 19, 2017. Study participants included sexually active patients aged 13 to 21 years at time of enrollment who were planning to or had already received the quadrivalent HPV vaccine (4vHPV) targeting HPV types 6, 11, 16, and 18. Pregnant and menstruating women were invited to return at a later date after resolution of their pregnancy or their menstrual period. No selection was done by race/ethnicity. The study sample excluded 630 consented participants who had not completed a baseline visit as of March 9, 2017. The study was approved by the Institutional Review Board at Icahn School of Medicine at Mount Sinai, Manhattan, New York, and written informed consent was obtained from all study participants and from guardians accompanying minors before enrollment. This study followed the Strengthening the Reporting of Observational Studies in Epidemiology (STROBE) reporting guideline.

### Study Questionnaires and Specimens

Study participants were followed up every 6 months until age 25 years, with collection of oral, cervical, and anal samples for HPV testing. Oral samples were collected by rinsing and gargling with an alcohol-based mouthwash for 30 seconds. Cervical samples collected by cytobrush and anal samples collected by a swab were also obtained for HPV testing at each study visit.

Information on lifetime and recent sexual behaviors, including number of vaginal, anal, and oral sex partners, was collected at enrollment and at each 6-month follow-up visit by self-reported questionnaire. History of other sexually transmitted diseases, pregnancies, short-term and long-term contraceptive use, tobacco smoking, and alcohol, marijuana, and illicit drug use were also collected at each study visit. Additional details on the study design and protocol are described elsewhere.^9^ All participants received a complete gynecological assessment, including cervical Papanicolaou test, during the initial visit and then annually once they reached age 21 years.

### HPV Testing

DNA was extracted from collected samples and tested for more than 40 HPV types known to infect mucosal tissue, including the anogenital area, by using a previously described protocol based on the MY09/MY11 polymerase chain reaction platform.^10^ The study sample size at the time of analysis included 1259 participants who had oral rinse samples with sufficient DNA for HPV testing, including concurrent amplification of a human β-globin gene fragment as a control.^11^

### Statistical Analysis

Characteristics of the study cohort were described using frequency distributions. Prevalence of HPV in oral samples was assessed for all types combined, individual oncogenic types, and 4vHPV vaccine types (HPV-6, HPV-11, HPV-16, and HPV-18) at baseline (enrollment visit), with exact binomial 95% CIs. Cross-sectional associations with detection of HPV DNA in oral samples were assessed by logistic regression after adjustment for participant age in years.

To study the association between oral HPV prevalence and risk associations with sexual behaviors, we fitted logistic regression models to assess the association of number of oral sexual partners, vaginal sexual partners, or both with oral HPV detection at enrollment. Additional models also assessed time since initiation of sexual activity. Models were adjusted for age in years, with further adjustment for statistically significant covariates, including concurrent detection of cervical HPV and time since initiation of sexual activity. Tests for trend for ordered categorical variables were conducted by including the variables in the model as ordinal values.

Cumulative incidence was estimated by using the Kaplan-Meier method for incident infections detected in oral samples collected at follow-up visits. Time to event was estimated by taking the midpoint of the interval preceding incident events. Participants who were HPV negative at enrollment and who did not have an infection until the last visit were censored at the last follow-up date. Statistical calculations were conducted using Stata version 14 (StataCorp, LLC), and statistical significance was assumed at the 2-tailed α = .05 level.

## Results

Of the 1259 participants, 638 (50.7%) were of African American descent, 569 (45.2%) were of Hispanic descent, 43 (3.4%) reported another race/ethnicity, and 9 (0.7%) were of unspecified race/ethnicity (Table 1). The median age at entry into the study was 18 (range, 13-21) years. At the time of enrollment, 878 (69.7%) had had at least 3 lifetime sex partners, with half reporting first sexual intercourse before age 16 years. The median (mode) age at first sexual activity was 14.8 (14) years, and 1161 (92.2%) reported having had oral sex.

**Table 1.** Cohort Characteristics at Baseline for 1259 Female Adolescents With Oral Rinse Samples Tested for HPV

Characteristic	Participants, No. (%)^a^
Age, y	
13-14	21 (1.7)
15-16	224 (17.8)
17-18	600 (47.7)
19-21	414 (32.9)
Race/ethnicity	
African American	638 (50.7)
Hispanic	569 (45.2)
Other	43 (3.4)
Unspecified	9 (0.7)
Lifetime No. of vaginal sex partners	
1	228 (18.2)
2	215 (17.2)
3-4	369 (29.5)
5-9	306 (24.4)
≥10	135 (10.8)
Oral sex	
None	98 (7.8)
Yes	
Received	135 (10.7)
Given	21 (1.7)
Both	1004 (79.9)
Lifetime No. of oral sex partners (given)	
0	233 (18.5)
1	334 (26.6)
2	252 (20.0)
3-4	289 (23.0)
5-9	112 (8.9)
≥10	38 (3.0)
Years since first sexual activity	
≤1	273 (21.7)
2	283 (22.5)
3	273 (21.7)
≥4	428 (34.1)
Marijuana use	
Nonuser	443 (35.2)
Former user	354 (28.1)
Current	451 (35.8)
Cigarette smoking	
Nonuser	727 (57.7)
Former user	56 (4.5)
Current	473 (37.6)
Cervical HPV status	
Negative	657 (53.6)
Positive	570 (46.5)

Abbreviation: HPV, human papillomavirus.

^a^ Numbers may not total 1259 because of missing data.

### Prevalence of Oral HPV Among Sexually Active Female Adolescents

**Figure 1.**
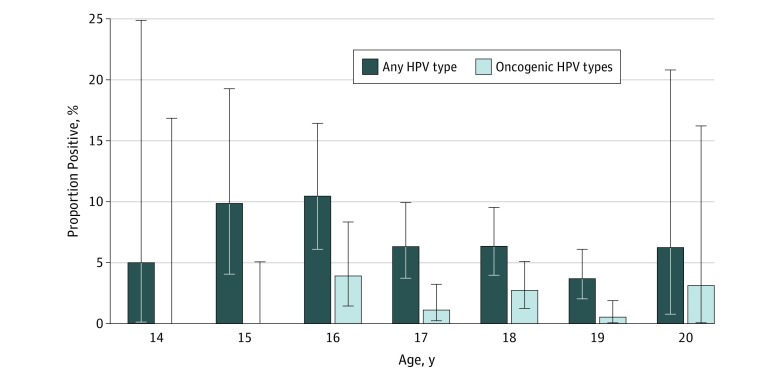
Prevalence of Oral Human Papillomavirus (HPV) Among Sexually Active Female Adolescents by Age at Enrollment Error bars indicate 95% CIs.

One oral rinse sample was obtained from each of the 1259 participants at enrollment. Human papillomavirus types were detected in the baseline oral rinse samples of 78 of the 1259 participants (6.2%; 95% CI, 4.9%-7.7%), with oncogenic HPV types detected in 21 (1.7%; 95% CI,1.0%-2.5%) and 4vHPV vaccine types (HPV-6, HPV-11, HPV-16, and HPV-18) detected in 8 participants (0.2%; 95% CI, 0.3%-1.3%). The greatest prevalence of oral HPV was detected at age 16 years for all types (16 of 153 [10.5%]) and oncogenic types (6 of 153 [3.9%]) (Figure 1).

### Associations With Sexual Activity and Other Putative Risk Factors

**Figure 2.**
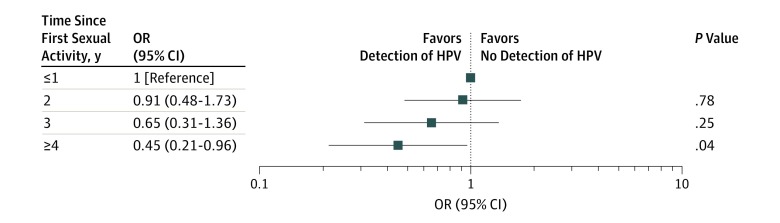
Association Between Detection of Human Papillomavirus (HPV) in the Oral Cavity and Time Since First Sexual Experience Odds ratios (ORs; with 95% CIs) for the associations between HPV detection in the oral cavity and time since first sexual experience reported at enrollment. Associations were adjusted for age and concurrent detection of cervical HPV.

Assessing associations of oral HPV detection with level of sexual activity, we observed no significant associations with giving or receiving oral sex compared with those who reported no oral sex after adjustment for age (Table 2). In contrast, we observed a significant decrease in oral HPV detection with years since first engaging in sexual activity, comparing participants who engaged in sex 4 or more years vs 1 year or less before testing (odds ratio [OR], 0.45; 95% CI, 0.21-0.96; *P* for trend = .03) (Figure 2).

**Table 2.** Cross-sectional Associations Between Participant Characteristics at Baseline and Detection of HPV in Oral Rinse Samples by the MY09/11 Polymerase Chain Reaction Protocol

Characteristic	Positive for Any HPV Type, No. (%)	Odds Ratio (95% CI)^a^
Age, y		
13-14	1 (4.8)	1 [Reference]
15-16	23 (10.3)	2.29 (0.3-18.0)
17-18	38 (6.3)	1.35 (0.2-10.4)
19-21	16 (3.9)	0.80 (0.1-6.4)
Race/ethnicity		
African American	37 (5.8)	1 [Reference]
Hispanic	38 (6.7)	1.18 (0.7-1.9)
Other	2 (4.7)	0.87 (0.2-3.7)
Unspecified	1 (11.1)	2.21 (0.3-18.3)
Lifetime No. of vaginal sex partners		
1	15 (6.6)	1 [Reference]
2	13 (6.0)	1.00 (0.5-2.2)
3-4	23 (6.2)	1.12 (0.6-2.2)
5-9	17 (5.6)	1.04 (0.5-2.2)
≥10	9 (6.7)	1.27 (0.5-3.0)
Oral sex		
None	9 (9.2)	1 [Reference]
Yes		
Received	9 (6.7)	0.75 (0.3-2.0)
Given	2 (9.5)	1.09 (0.2-5.5)
Both	58 (5.8)	0.74 (0.3-1.6)
Lifetime No. of oral sex partners (given)		
0	18 (7.7)	1 [Reference]
1	22 (6.6)	0.94 (0.5-1.8)
2	16 (6.3)	0.95 (0.5-1.9)
3-4	17 (5.9)	0.90 (0.4-1.8)
5-9	4 (3.6)	0.56 (0.2-1.7)
≥10	1 (2.6)	0.39 (0.1-3.0)
Years since first sexual activity		
≤1	23 (8.4)	1 [Reference]
2	24 (8.5)	1.09 (0.6-2.0)
3	16 (5.9)	0.78 (0.4-1.6)
≥4	15 (3.5)	0.49 (0.2-1.0)
Marijuana use		
Nonuser	33 (7.5)	1 [Reference]
Former user	26 (7.3)	0.98 (0.6-1.7)
Current	19 (4.2)	0.55 (0.3-1.1)
Cigarette smoking		
Nonuser	52 (7.2)	1 [Reference]
Former user	3 (5.4)	0.73 (0.2-2.4)
Current	23 (4.9)	0.68 (0.4-1.1)
Cervical HPV status		
Negative	29 (4.4)	1 [Reference]
Positive	43 (7.5)	1.80 (1.1-2.9)

Abbreviation: HPV, human papillomavirus.

^a^ Odds ratios and 95% CIs are estimated by logistic regression after adjustment for age in years.

Although marijuana use and cigarette smoking appeared to be inversely associated with detection of HPV in the oral cavity, these were not significant after adjustment for age and time since initiation of sexual activity (marijuana use: OR, 0.77; 95% CI, 0.5-1.3; cigarette smoking: OR, 0.76; 95% CI, 0.5-1.3). The most consistent risk factor associated with detection of HPV in the oral cavity was detection of HPV in the cervical sample collected at the same visit.

### Association of Vaccination With Detection of HPV in the Oral Cavity

At enrollment, 192 of the 1259 study participants (15.3%) had not received the 4vHPV vaccine. eFigure 1 in the Supplement shows the type-specific prevalence of HPV types targeted by the current HPV vaccines, as well as other oncogenic HPV types among vaccinated and unvaccinated female adolescents. When we looked at the prevalence of individual 4vHPV vaccine types (HPV-6, HPV-11, HPV-16, and HPV-18), we found lower detection of HPV-16 among vaccinated compared with unvaccinated women (1 of 1067 vs 2 of 192), although the differences were not significant by Fisher exact test (*P* = .06).

To further investigate the association of vaccination with detection of any 4vHPV types, we used a multivariable logistic regression approach to compare vaccinated with unvaccinated participants after adjustment for age, years since first sexual activity, and concurrent detection of 4vHPV types in the cervix (Table 3). Prevalence of 4vHPV types among all vaccinated participants was 83% lower compared with unvaccinated participants (odds ratio [OR], 0.17; 95% CI, 0.04-0.68). The association was somewhat attenuated but remained protective after adjustment for concurrent detection of cervical 4vHPV types (OR, 0.20; 95% CI, 0.04-1.00).

**Table 3.** Association Between Vaccine Status at Enrollment and Detection of Quadrivalent HPV Vaccine Types in the Oral Cavity^a^

Vaccine Status	No. of Participants (% HPV Positive)	Odds Ratio (95% CI)^b^	Odds Ratio (95% CI)^c^
No. of doses			
0	192 (2.1)	1 [Reference]	1 [Reference]
>1	1067 (0.4)	0.17 (0.04-0.68)	0.20 (0.04-0.998)

Abbreviation: HPV, human papillomavirus.

^a^ Quadrivalent HPV vaccine types include HPV-6, HPV-11, HPV-16, and HPV-18.

^b^ Odds ratios and 95% CIs were estimated by logistic regression after adjustment for age and years since first sexual activity.

^c^ Additional adjustment for concurrent cervical detection of quadrivalent HPV vaccine types.

### Continued Risk of HPV

When we assessed incident detection of oral HPV among vaccinated women in our cohort, rates of infection were low overall, ranging from 0.05 (95% CI, 0.01-0.20) to 0.46 (95% CI, 0.29-0.73) HPV infections per 100 person-years (eFigure 2 in the Supplement). Eighty-eight percent of oral HPV cases that were detected cleared within 12 months, reflecting the highly transient nature of these infections. Nonetheless, 2 participants presented with persistent (>1 year) oral HPV (1 with HPV-6/-11 and 1 with HPV-16), suggesting a continued burden in some individuals; no oral or cervical disease was found in these individuals.

## Discussion

Recent data from NHANES showed that detection of oral HPV is relatively low among women (compared with men), ranging between 2% and 5%.^4^ In the present study, we observed an overall oral HPV prevalence of 6.2% for HPV types among sexually active female adolescents in New York City.

Our data do not provide strong support of a sexual transmission model for oral HPV acquisition, although detection was highest soon after initiation of sexual activity.^12,13^ We observed a decrease in detection of HPV in the oral cavity with years since the first sexual activity, potentially reflecting the highly transient nature of HPV infection.^14^ Concurrent detection of cervical HPV was itself significantly associated with detection of HPV in the oral cavity, suggesting that the anogenital region may be a source and that transmission could involve digital transfer or autoinoculation.^15,16^

Although increased sexual activity has been identified as an important risk factor for oral HPV,^17^ there is little information about associations in sexually active adolescents. Consistent with previous studies,^4,18,19^ we report that any sexual activity, not just oral sex, may be associated with oral HPV detection. In comparison with previous studies, however, we observed no association between the likelihood of HPV detection in the oral cavity and number of oral sex partners, vaginal sex partners, or both.^20^ This may be owing in part to the lack of a reference group of individuals who had not engaged in sexual activity in our study cohort.^21^

### Strengths and Limitations

Our study population had a greater median number of sexual partners and an earlier initiation of sexual activity compared with previously reported cohorts, including the vaccine trial participants.^22^ The current cohort was also more likely to present with an oncogenic HPV type or vaccine type in the oral cavity and cervix compared with other groups.^10^ Despite these factors, we observed a significant vaccination benefit reflected by the lower prevalence of HPV vaccine types (HPV-6, HPV-11, HPV-16, and HPV-18) in the oral cavity among vaccinated vs unvaccinated female adolescents. Results from the bivalent HPV vaccine trial conducted among women aged 18 to 25 years showed a vaccine efficacy of 93% against detection of oral HPV-16 and HPV-18 four years after vaccination.^7^ In comparison, Chaturvedi et al^23^ showed an 88% reduction in prevalence of oral HPV-16 and HPV-18 when assessing differences by self-reported vaccine status among participants in NHANES. Our estimates of vaccine benefit among sexually active female adolescents are in line with these population estimates. However, given the low prevalence of oral HPV and the inclusion of vaccinated participants, our study had insufficient statistical power to assess associations with individual HPV types.

A number of studies have shown HPV-16 to be the main HPV type associated with oropharyngeal cancers in the United States.^21,24^ Although we observed a decreased prevalence of HPV-16 and other 4vHPV vaccine types among vaccinated female adolescents, we also detected new HPV-16 infections in the oral samples at a rate of 0.2 per 100 person-years. In addition, repeated detection of 4vHPV vaccine types in oral rinse samples across a period of more than 1 year was observed in at least 2 participants, suggesting a subset of individuals with persistent infections that were likely acquired before vaccination. We also observed similar or higher incidence for other oncogenic nonvaccine HPV types (ie, HPV-33, HPV-35, HPV-39, HPV-51, HPV-58, and HPV-66)^25^; continued research is needed to determine whether the additional types targeted by the new 9-valent vaccine will also decrease.^26^

## Conclusions

Overall, we found that detection of HPV in the oral cavity is not uncommon in sexually active female adolescents, but it decreases with age and time since the initiation of sexual activity. Although our study population had a greater median number of sexual partners and a younger age at sexual initiation compared with previous studies, we observed a significant vaccination benefit reflected by the lower prevalence of 4vHPV vaccine types. Our findings suggest that concurrent detection of an HPV infection in the cervix may be a risk factor for detection of HPV in the oral cavity.

## Notes

Supplement. **eFigure 1.** Prevalence of Oral HPV by Vaccine Status in Sexually Active Adolescent Females **eFigure 2.** Oral HPV Incidence Rates Post-Vaccination in Sexually Active Adolescent Females
