# Supplementary material for: Risk of Oral Human Papillomavirus Infection Among Sexually Active Female Adolescents Receiving the Quadrivalent Vaccine
Source: JAMA Netw Open. 2019 Oct 25;2(10):e1914031. doi: 10.1001/jamanetworkopen.2019.14031 (PMC6822084; doi:10.1001/jamanetworkopen.2019.14031)

Supplementary Online Content

Schlecht NF, Masika M, Diaz A, et al. Risk of oral human papillomavirus infection among sexually active female adolescents receiving the quadrivalent vaccine. *JAMA Netw Open*. 2019;2(10):e1914031. doi:10.1001/jamanetworkopen.2019.14031

eFigure 1. Prevalence of Oral HPV by Vaccine Status in Sexually Active Adolescent Females

eFigure 2. Oral HPV Incidence Rates Post-Vaccination in Sexually Active Adolescent Females

This supplementary material has been provided by the authors to give readers additional information about their work.

eFigure 1. Prevalence of Oral HPV by Vaccine Status in Sexually Active Adolescent Females

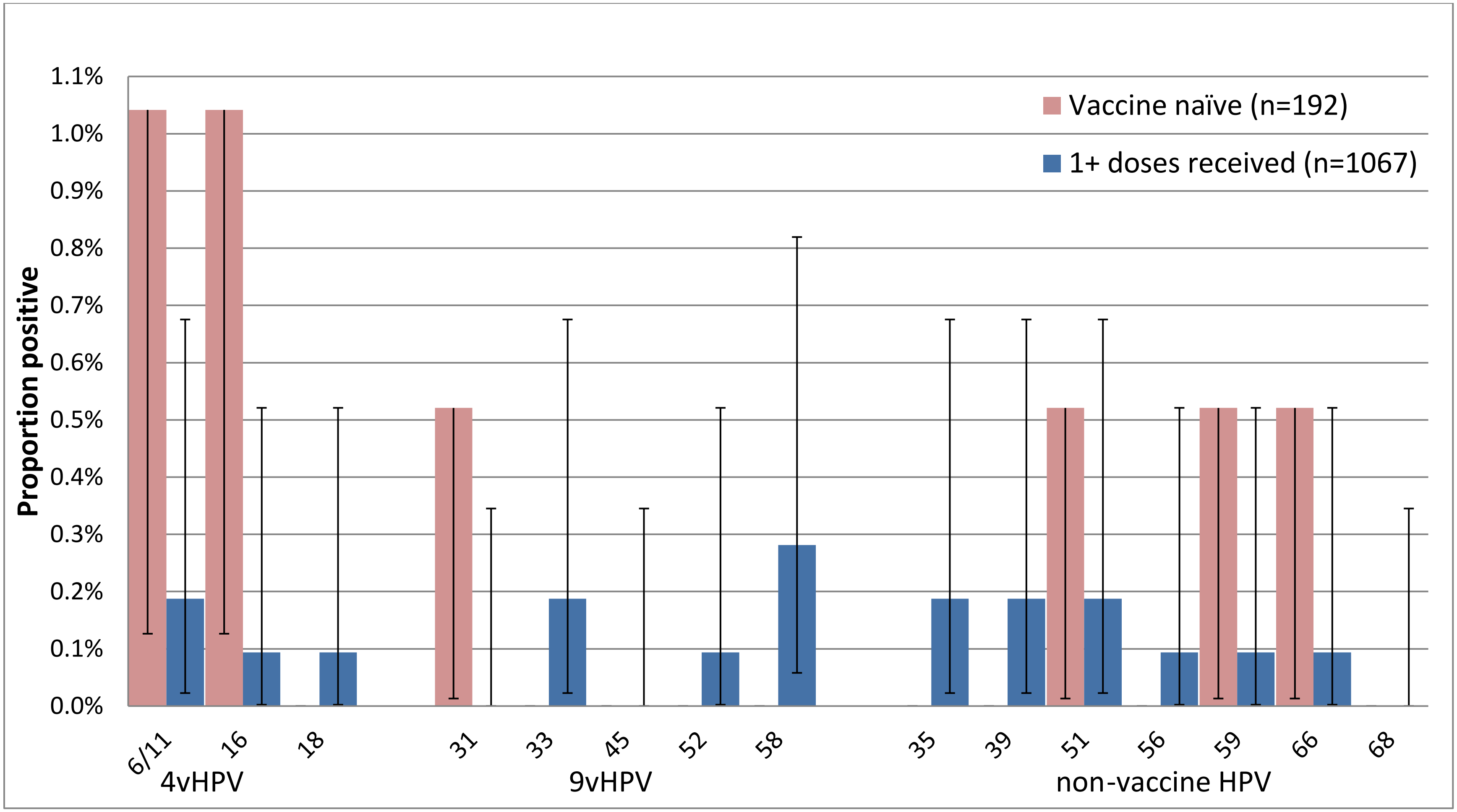

eFigure 2. Oral HPV Incidence Rates Post-Vaccination in Sexually Active Adolescent Females

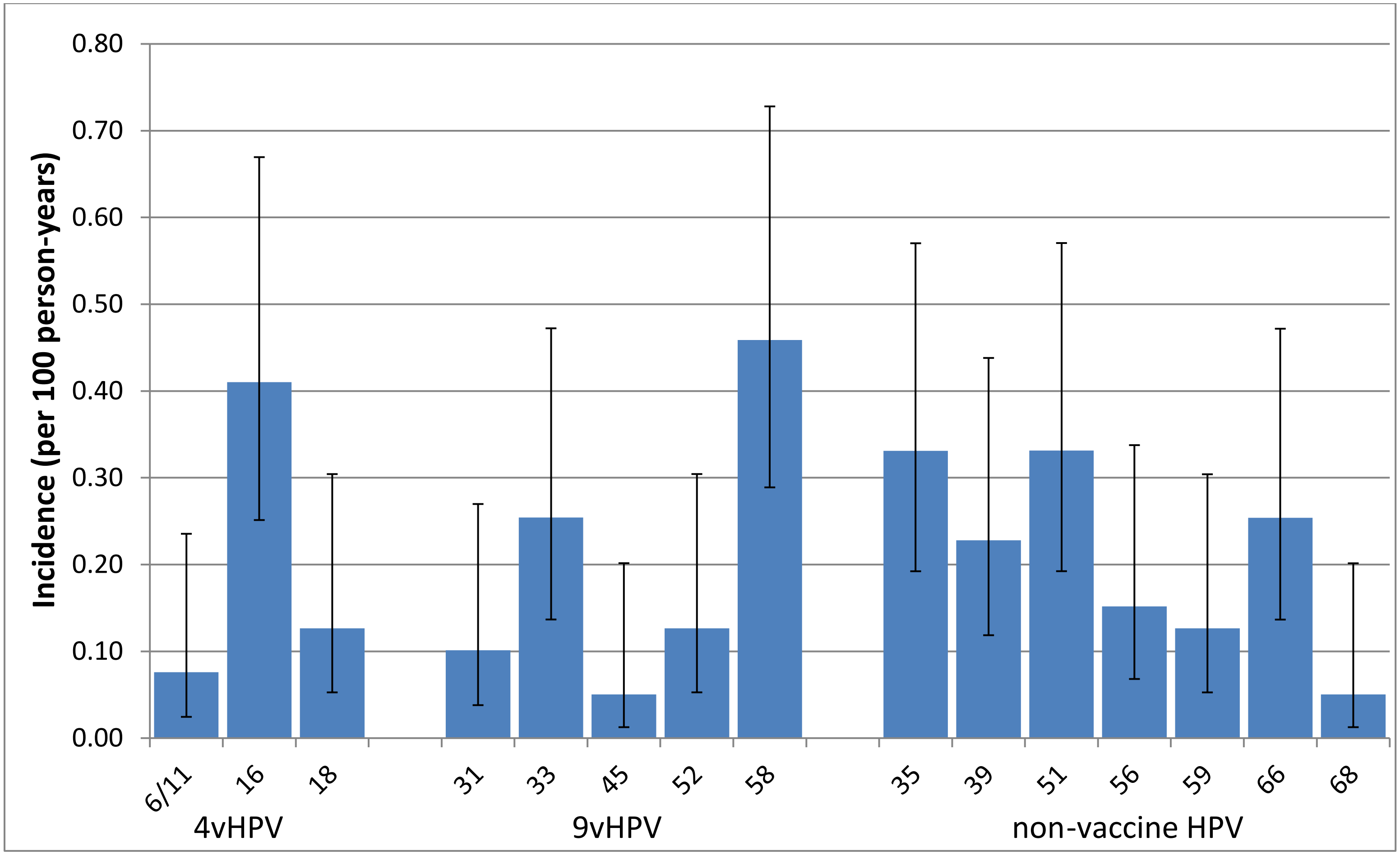

Supplement: Supplement. — eFigure 1. Prevalence of Oral HPV by Vaccine Status in Sexually Active Adolescent Females eFigure 2. Oral HPV Incidence Rates Post-Vaccination in Sexually Active Adolescent Females [file jamanetwopen-2-e1914031-s001.pdf]
